# Supplementary material for: Artificial intelligence for early detection of renal cancer in computed tomography: A review
Source: Camb Prism Precis Med. 2022 Nov 11;1:e4. doi: 10.1017/pcm.2022.9 (PMC10953744; doi:10.1017/pcm.2022.9)
Supplement: Supplementary file 1 [file S2752614322000096sup001.docx]

| **Title** | **Authors** | **Year** | **Included in Review** | **Description** |
| --- | --- | --- | --- | --- |
| A Coarse-to-Fine Framework for the 2021 Kidney and Kidney Tumor Segmentation Challenge. | Zhao, Z., H. Chen, and L. Wang | 2022 | Y | Renal Segmentation |
| A collaborative computer aided diagnosis (C-CAD) system with eye-tracking, sparse attentional model, and deep learning | Khosravan, N., et al. | 2019 | Y | Broader AI Literature |
| A comparative study of contrast-enhanced ultrasound and contrast-enhanced CT for the detection and characterization of renal masses | Fang, L, et al. | 2021 | N | - |
| A convnet for the 2020s | Liu, Z., et al | 2022 | Y | ImageNet Submission |
| A Multi-Task Convolutional Neural Network for Renal Tumor Segmentation and Classification Using Multi-Phasic CT Images. | Pan, T., et al. | 2019 | Y | Renal Classification |
| A Precision Diagnostic Framework of Renal Cell Carcinoma on Whole-Slide Images using Deep Learning | Wu, J, et al. | 2021 | N | - |
| An attempt at beating the 3D U-Net. | Isensee, F. and K.H. Maier-Hein. | 2019 | Y | Renal Segmentation |
| An Image is Worth 16x16 Words: Transformers for Image Recognition at Scale | Dosovitskiy, A., et al. | 2020 | Y | ImageNet Submission |
| Angiomyolipoma of the Kidneys: Current Perspectives and Challenges in Diagnostic Imaging and Image-Guided Therapy | Wu, J, et al. | 2019 | N | - |
| Angiomyolipoma with minimal fat: differentiation from clear cell renal cell carcinoma and papillary renal cell carcinoma by texture analysis on CT images | Yan, L., et al. | 2015 | N | - |
| Aorta-aware GAN for non-contrast to artery contrasted CT translation and its application to abdominal aortic aneurysm detection | Hu, T., et al. | 202 | Y | Broader AI Literature |
| Assessment of tumor heterogeneity by CT texture analysis: can the largest cross-sectional area be used as an alternative to whole tumor analysis? | Ng, F., et al. | 2013 | N | - |
| Attention is All You Need | Vaswani, A., et al. | 2017 | Y | ImageNet Submission |
| Automated classification of solid renal masses on contrast-enhanced computed tomography images using convolutional neural network with decision fusion | Zabihollahy, F., et al. | 2020 | Y | Renal Classification |
| Automated Detection and Classification of Oral Lesions Using Deep Learning for Early Detection of Oral Cancer | Welikala, R.A., et al. | 2020 | Y | Broader AI Literature |
| Automated Kidney Tumor Segmentation with Convolution and Transformer Network | Shen, Z., et al. | 2022 | N | - |
| Automatic Kidney Lesion Detection for CT Images Using Morphological Cascade Convolutional Neural Networks | Zhang, H., et al. | 2019 | Y | Renal Classification |
| Can High-Attenuation Renal Cysts Be Differentiated from Renal Cell Carcinoma at Unenhanced CT? | Jonisch, A., et al. | 2007 | N | - |
| Can quantitative CT texture analysis be used to differentiate fat-poor renal angiomyolipoma from renal cell carcinoma on unenhanced CT images? | Hodgdon, T., et al. | 2015 | Y | Renal Classification |
| Can whole-tumor radiomics-based CT analysis better differentiate fat-poor angiomyolipoma from clear cell renal cell caricinoma: compared with conventional CT analysis? | Ma, Y., et al. | 2020 | Y | Renal Classification |
| Characterization of Small Solid Renal Lesions: Can Benign and Malignant Tumors Be Differentiated With CT? | Millet, I., et al. | 2011 | N | - |
| Circle Representation for Medical Object Detection | Nguyen, E.H., et al. | 2022 | Y | Broader AI Literature |
| Classification of renal tumour using convolutional neural networks to detect oncocytoma | Pedersen, M., et al. | 2020 | Y | Renal Classification |
| Comparison of Contrast-Enhanced Multiphase Renal Protocol CT Versus MRI for Diagnosis of Papillary Renal Cell Carcinoma | Dilaruo, M., et al. | 2016 | N | - |
| Co-scale conv-attentional image transformers | Xu, W., et al. | 2021 | Y | ImageNet Submission |
| Creation of synthetic contrast-enhanced computed tomography images using deep neural networks to screen for renal cell carcinoma. | Sassa, N., et al. | 2022 | Y | Broader AI Literature |
| CT diagnosis of renal angiomyolipoma: the importance of detecting small amounts of fat | Bosniak, M., et al. | 1988 | N | - |
| Current Concepts in the Diagnosis and Management of Renal Cell Carcinoma: Role of Multidetector CT and Three-dimensional CT | Sheth, S., et al. | 2001 | N | - |
| Deep Ensemble Learning Network for Kidney Lesion Detection | Xiong, Z., et al. | 2019 | Y | Renal Classification |
| Deep feature classification of angiomyolipoma without visible fat and renal cell carcinoma in abdominal contrast-enhanced CT images with texture image patches and hand-crafted feature concatenation. | Lee, H., et al. | 2018 | Y | Renal Classification |
| Deep learning based classification of solid lipid-poor contrast enhancing renal masses using contrast enhanced CT | Oberai, A., et al. | 2020 | Y | Renal Classification |
| Deep learning for end-to-end kidney cancer diagnosis on multi-phase abdominal computed tomography | Uhm, K.-H., et al. | 2021 | Y | Renal Classification |
| Deep learning methods to generate synthetic CT from MRI in radiotherapy: A literature review | Boulanger, M., et al. | 2021 | N | - |
| Deep Learning to Improve Breast Cancer Early Detection on Screening Mammography | Shen, L., at al. | 2019 | N | - |
| Deep Residual Networks for Image Recognition | He, K., et al. | 2016 | Y | ImageNet Submission |
| Deeplesion: automated mining of large-scale lesion annotations and universal lesion detection with deep learning | Yan, K., et al. | 2018 | Y | Broader AI Literature |
| Development and Validation of a Deep-learning Model to Assist With Renal Cell Carcinoma Histopathologic Interpretation | Fenstermaker, M., et al. | 2020 | Y | Renal Classification |
| Diagnosis of Sarcomatoid Renal Cell Carcinoma With CT: Evaluation by Qualitative Imaging Features and Texture Analysis | Schieda, N., et al. | 2015 | Y | Renal Classification |
| Diagnostic Accuracy of Unenhanced CT Analysis to Differentiate Low-Grade From High-Grade Chromophobe Renal Cell Carcinoma | Schieda, N., et al. | 2018 | Y | Renal Classification |
| Differentiation of Predominantly Solid Enhancing Lipid-Poor Renal Cell Masses by Use of Contrast-Enhanced CT: Evaluating the Role of Texture in Tumor Subtyping | Varghese, B.A., et al. | 2018 | Y | Renal Classification |
| Differentiation of renal angiomyolipoma without visible fat from small clear cellrenal cell carcinoma by using specificregion of interest on contrast-enhanced CT:a new combination of quantitative tools. | Wang, X., Ge Song, and H. Jiang | 2021 | Y | Renal Classification |
| Differentiation of Small (≤ 4 cm) Renal Masses on Multiphase Contrast-Enhanced CT by Deep Learning | Tanaka, T., et al. | 2020 | Y | Renal Classification |
| Dyefreenet: Deep Virtual Contrast CT Synthesis | Liu, J., et al. | 2020 | Y | Broader AI Literature |
| Effect of phase of enhancement on texture analysis in renal masses evaluated with non-contrast-enhanced, corticomedullary, and nephrographic phase–enhanced CT images | Nguyen, K., et al. | 2021 | N | - |
| End-to-end lung cancer screening with three-dimensional deep learning on low-dose chest computed tomography | Ardila, D., et al. | 2019 | Y | Broader AI Literature |
| Facial Landmark Detection by Deep Multi-task Learning | Zhang, Z., et al. | 2014 | N | - |
| Free-form tumor synthesis in computed tomography images via richer generative adversarial network | Jin, Q., et al. | 2018 | Y | Broader AI Literature |
| Image-to-Image Translation with Conditional Adversarial Networks | Isola, P., et al. | 2017 | Y | Broader AI Literature |
| Importance of phase enhancement for machine learning classification of solid renal masses using texture analysis features at multi-phasic CT | Schieda, N., et al. | 2020 | N | - |
| Machine Learning Approach to Predict the Probability of Recurrence of Renal Cell Carcinoma After Surgery: Prediction Model Development Study | Hyung Min, K. et al. | 2021 | N | - |
| Machine learning-based mortality prediction of patients undergoing cardiac resynchornisation therapy: the SEMMELWEIS score | Tokodi, M., et al. | 2020 | N | - |
| Machine learning-based mortality prediction: how to be connected to daily clinical practice? | Kim, W. and Kim, J-T. | 2020 | N | - |
| Machine learning-based quantitative texture analysis of CT images of small renal masses: Differentiation of angiomyolipoma without visible fat from renal cell carcinoma. | Feng, Z., et al. | 2018 | Y | Renal Classification |
| MB-FSGAN: Joint segmentation and quantification of kidney tumor on CT by the multi-branch feature sharing generative adversarial network | Ruan, Y., et al. | 2020 | Y | Broader AI Literature |
| Multidetector Computed Tomography in the Evaluation of Renal Masses--A Prospective Study from Ambala, India. | Kuchhal, A., et al. | 2021 | N | - |
| Multi-level Wavelet-CNN for Image Restoration | Liu, P., et al. | 2018 | Y | Broader AI Literature |
| Multimodal Deep Learning for Prognosis Prediction in Renal Cancer | Schulz, S., et al. | 2021 | N | - |
| Multimodality imaging evaluation of pseudotumors in chronic renal dysfunction: exposing the masquerade! | Goyal, A., et al. | 2021 | N | - |
| Multi-Task Learning as Multi-Objective Optimization | Sener, O. and Koltun, V. | 2018 | N | - |
| Nnu-Net: a self-configuring method for deep learning-based biomedical image segmentation. | Isensee, F., et al. | 2021 | Y | Renal Segmentation |
| Objective risk stratification of prostate cancer using machine learning and radiomics applied to multiparametric magnetic resonance images | Varghese, B.A., et al. | 2019 | N | - |
| Ovarian cancer detection using optical coherence tomography and convolutional neural networks | Schwartz, D., et al. | 2022 | N | - |
| Pan-Renal Cell Carcinoma classification and survival prediction from histopathology images using deep learning | Tabibu, S., P. K. Vinod, and C.V. Jawahar | 2019 | Y | Renal Classification |
| Predicting sex from retinal fundus photographs using automated deep learning | Korot, E., et al. | 2021 | N | - |
| Prediction of Benign and Malignant Solid Renal Masses: Machine Learning-Based CT Texture Analysis | Erdim, C., et al. | 2020 | Y | Renal Classification |
| Quantitative Analysis of Multiphase Contrast-Enhanced CT Images: A Pilot Study of Preoperative Prediction of Fat-Poor Angiomyolipoma and Renal Cell Carcinoma | Tang, Z., et al. | 2020 | N | - |
| Radiologic-Radiomic Machine Learning Models for Differentiation of Benign and Malignant Solid Renal Masses: Comparison With Expert-Level Radiologists | Sun, X.-Y., et al. | 2020 | Y | Renal Classification |
| Radiomics Applications in Renal Tumor Assessment: A Comprehensive Review of the Literature | Suarez-Ibarrola, R., et al. | 2020 | N | - |
| Relative Enhancement Ratio of Portal Venous Phase to Unenhanced CT in the Diagnosis of Lipid-poor Adrenal Adenomas | Nagayama, Y., et al. | 2021 | N | - |
| Renal angiomyolipoma without visible fat: Can we make the diagnosis using CT and MRI? | Lim, R., et al. | 2018 | N | - |
| Renal cell carcinoma attenuation values on unenhanced CT: importance of multiple, small region-of-interest measurements | McGahan, J., et al. | 2017 | N | - |
| Renal cell carcinoma: attenuation values on unenhanced CT | Pooler, B., et al. | 2012 | N | - |
| Renal cell carcinoma: correlation of CT findings with nuclear morphologic grading in 100 tumors | Birnbaum, B., et al. | 1994 | N | - |
| Rethinking the Inception Architecture for Computer Vision | Szegedy, C., et al. | 2016 | Y | ImageNet Submission |
| Small (<4 cm) clear cell renal cell carcinoma: correlation between CT findings and histologic grade | Choi, S., et al. | 2016 | N | - |
| Solid Renal Cell Carcinoma Measuring Water Attenuation (−10 to 20 HU) on Unenhanced CT | Schieda, N., et al. | 2015 | N | - |
| Solid Small Renal Mass Without Gross Fat: CT Criteria for Achieving Excellent Positive Predictive Value for Renal Cell Carcinoma | Park, S., et al. | 2018 | N | - |
| Swin Transformer: Hierarchical Vision Transformer using Shifted Windows | Liu, Z., et al. | 2021 | Y | ImageNet Submission |
| Synthetic contrast enhancement in cardiac CT with Deep Learning | Santini, G., et al. | 2018 | Y | Broader AI Literature |
| T1a renal cell carcinoma on unenhanced CT: analysis of detectability and imaging features | Gobara, A., et al. | 2019 | N | - |
| Tensor Programs V: Tuning Large Neural Networks via Zero-Shot Hyperparameter Transfer | Yang, G., et al. | 2021 | Y | Broader AI Literature |
| Textural differences between renal cell carcinoma subtypes: Machine learning-based quantitative computed tomography texture analysis with independent external validation | Kocak, B., et al. | 2018 | Y | Renal Classification |
| The Classification of Renal Cancer in 3-Phase CT Images Using a Deep Learning Method. | Han, S., Sung Il Hwang, and H.J. Lee | 2019 | Y | Renal Classification |
| The Value of ‘‘Liver Windows’’ Settings in the Detection of Small Renal Cell Carcinomas on Unenhanced Computed Tomography - liver windows refers to the HU value range for the liver. | Sahi, K., et al. | 2014 | N | - |
| The value of quantitative CT texture analysis in differentiation of angiomyolipoma without visible fat from clear cell renal cell carcinoma on four-phase contrast-enhanced CT images | You, M-W., Kim, N., and Choi, H-J. | 2019 | N | - |
| The value of unenhanced helical computerized tomography in the management of acute flank pain | Dalrymple, N., et al. | 1998 | N | - |
| Transunet: Transformers Make Strong Encoders for Medical Image Segmentation | Chen, J., et al. | 2021 | Y | Broader AI Literature |
| Triage-driven diagnosis for early detection of esophageal cancer using deep learning | Gehrung, M., et al. | 2021 | Y | Broader AI Literature |
| Unenhanced CT for the detection of renal cell carcinoma: effect of tumor size and contour type | Jung, S., et al. | 2014 | N | - |
| Unenhanced CT Texture Analysis of Clear Cell Renal Cell Carcinomas: A Machine Learning-Based Study for Predicting Histopathologic Nuclear Grade | Kocak, B., et al. | 2019 | N | - |
| Unenhanced helical computerized tomography for the evaluation of patients with acute flank pain | Vieweg, J., et al. | 1998 | N | - |
| U-Net: Convolutional Networks for Biomedical Image Segmentation | Ronneberger, O., P. Fischer, and T. Brox | 2015 | Y | Broader AI Literature |
| UNETR: Transformers for 3D Medical Image Segmentation | Hatamizadeh, A., et al. | 2022 | Y | Broader AI Literature |
| Very deep convolutional networks for large-scale image recognition | Simonyan, K. and A. Zisserman | 2014 | Y | ImageNet Submission |
| Which Tasks Should Be Learned Together in Multi-task Learning? | Standley, T., et al. | 2020 | Y | Broader AI Literature |
